# A novel method for measuring heat injury in leaves provides insights into the sequence of processes of heat injury development

**DOI:** 10.1186/s13007-025-01404-6

**Published:** 2025-07-01

**Authors:** Clara Bertel, Gilbert Neuner

**Affiliations:** Department of Botany, University of Innsbruck, Sternwartestr. 15, 6020 Innsbruck, Austria

**Keywords:** DSC, Heat tolerance, Heat stress, Mountain plants, PS II thermotolerance

## Abstract

**Background:**

Global warming is currently occurring at a rapid rate and is having a particularly severe impact on plants, which, as sessile organisms, have a limited ability to escape high temperatures. This requires a better understanding of the thermal limits for different plant species and a better understanding of the processes involved in the development of heat injury in plant leaves. Heat injury results from multiple processes and occurs at the molecular level, involving increased membrane fluidity, lipid peroxidation, and protein aggregation and denaturation.

**Results:**

We have tested whether the DSC method allows the detection of heat-induced denaturation and aggregation of molecules in intact leaves. During controlled heating a consistent and repeatable pattern was observed in the DSC plot, from which critical heat thresholds could be derived. These critical temperatures were in good agreement with the temperatures determined using classical methods and also clearly mark the thermal limits of molecular structures. The advantage of the DCS method is the precise, rapid and easy detection of heat thresholds. Finally, taken all thresholds together, we can draw a better image of the sequence of events associated with heat injury in plant leaves: heat injury begins with membrane leakage and continues with protein denaturation and aggregation at high (sublethal, lethal) temperatures.

**Conclusion:**

Since heat injury results from multiple processes, a holistic understanding requires the acquisition of parameters indicative of different processes. The presented DSC method, which allows the detection of denaturation and aggregation of cellular compounds, therefore complements well the classical methods that reflect photosynthetic impairment and whole leaf tissue damage. The new simple and rapid method requires only a minimal amount of leaf material and allows rapid collection of data on damaging temperatures for different plants, which is particularly important in the face of rapidly progressing climatic changes.

**Supplementary Information:**

The online version contains supplementary material available at 10.1186/s13007-025-01404-6.

## Background

Heat damage occurs at the molecular level. It is associated with an increased fluidity of membranes, lipid peroxidation, and protein denaturation and aggregation [34, 42], all of which affect the integrity of cellular structures, including organelles, the cytoskeleton, and membrane functions [43]. Heat damage can occur at the level of cell membranes surrounding symplasts, but also at the level of organelle membranes, in case of the chloroplast affecting photosynthetic reactions. Within the phospholipid bilayer of membranes various proteins are embedded (fluid mosaic model, [37]). It is known that fluidity and thermostability of membranes can be adjusted by the chain length and degree of unsaturation or the integration of sterols and therefore the composition of membranes of plants (and other organisms) also differ with regard to their adaptation to the temperature climate [36]. Lipids are selectively embedded in the membrane bilayer. A direct consequence of detrimental heat is the denaturation of proteins and their aggregation and an increase in fluidity of membrane lipids [42]. Indirect and slower heat injuries occur due to the inactivation of enzymes in chloroplasts and mitochondria, disturbance of the organization of microtubules, production of toxic components and ROS [42]. However, the sequence of these events leading to heat damage is poorly understood.

When exposed to heat, molecular structures are changed, which causes either release or consumption of heat. This can be measured by calorimetry as a function of temperature or time by differential scanning calorimetry (DSC). In DSC, the difference in heat flow between a sample and a reference is measured. This allows the detection of molecular phase transitions by measuring heat produced (exothermic reactions) or absorbed (endothermic reactions) as a function of temperature. DSC is used for many applications in a wide range of industries, such as polymers, as well as for basic research in chemistry. Examples include the determination of glass transitions and the study of chemical reactions, melting and crystallisation behaviour. Usually, pure materials or solutes are measured. Studies exist about phase transition of isolated components, such as cell walls [22], lipids and biomembranes [20] including thylakoid membranes [5, 17, 18], protein denaturation and thermal stability of proteins [8, 12, 23, 25, 41], nucleic acid [38], polysaccharides [6, 48] and interactions of isolated compounds (e.g. [14]).

In case of isolated substances, temperatures of phase transitions can depend on the ionic strength of the medium [5], the concentration of isolated compounds, pH, and the surrounding, in which the compounds are embedded (e.g. dissolved or packed within a tissue, [31]). Only, recently, DSC was successfully employed to study the freezing dynamics in whole leaves [32, 40]. To our knowledge, DSC has never been used to measure heat-emitting or heat-consuming processes during controlled heating of intact leaves in order to determine critical thresholds for heat induced disintegration of biomolecules. The heat stability of molecular components in intact leaves must be considered to be of ecological relevance.

How heat induced disintegration of biomolecules relates to heat tolerance is unknown. Heat thresholds for leaves are usually measured by two contrasting methods: (1) in the classical heat tolerance test [16] leaves are immediately exposed to different target heat temperatures for 30 min and viability is assessed after development of heat damage (~ 4–7 days). The degree of heat damage can be measured by various methods: electrolyte leakage, chlorophyll fluorescence (maximum PSII efficiency, Fv/Fm) or visual assessment of the percentage of necrotic leaf area (e.g. [26]). (2) Secondly, the temperature dependent change in basic chlorophyll fluorescence (F_0_), T-F0 technique [2] can be used to measure the high temperature threshold of PSII, which is the most heat susceptible sub-process of photosynthesis. Both methods provide critical thresholds for heat damage, but these can differ widely by several °C [2, 29].

The investigation of critical heat thresholds has become a hot topic in plant ecological research due to the current and future impacts of climate change on plants. Along with a gradual increase in mean air temperature of 2.8 to 5.5 °C till 2100 (IPCC AR6 [10]), climate change is predicted to increase the intensity and frequency of heatwaves [30]. During the last two decades across Europe the days with extreme heat have more than tripled and hot extremes have warmed by 2.3 °C from 1958–2018 [24]. Global climate change may push plants closer to their physiological limits, depending on their ability to tolerate and adapt to heat, or migrate to cooler habitats—both rapid adaptation and migration appear to be limited [1, 4, 11, 13]. In the face of climate change, knowledge of critical heat thresholds and development of new screening methods is essential.

Measuring heat tolerance can be challenging as several processes have been associated with heat damage. Thus, the aims of this study were to (1) apply differential scanning calorimetry to determine critical heat thresholds of leaves and (2) to better understand the sequence of processes leading to heat-induced damage and their potential reversibility. We present a new application to test heat tolerance in intact leaves that is highly precise, very easy and fast to perform, requires a minimal amount of sample material and allows a wide range of experimental applications. Using multiple leaves from six mountain species we measured heat flow by DSC during constant heating from 25–65 °C and compared the results to parallel measurements of heat tolerance by use of classical heat tests and T-F0 curves. Specifically, we tested (1) whether the observed pattern is reproducible in different leaves of the same species, (2) whether it is valid in different species adapted to thermally different habitats. (3) We then defined parameters that can be easily derived in all DSC-plots and (3) compared them with heat thresholds whose physiological/biological significance is widely understood.

## Materials and methods

### Study species and sampling

We conducted our study using mountain plants, as mountains provide a wide range of thermal microhabitats at small spatial scale [15]. A set of common and native species was selected representing different growth forms, evolutionary ancestry, and adaptations to different temperature and habitat conditions (Supplemental Table S1). Healthy and fully mature leaves were sampled in 2024 (and additionally in 2023 for *Rhododendron ferrugineum* for comparison between years). For *R. ferrugineum* and *Vaccinium vitis-idea,* which have perennial evergreen leaves, leaves that were older than one year were selected. For the other species, which only develop their leaves in the current season, fully mature leaves were sampled. Depending on the growth habit, small branches, whole rosettes or the whole leaves with petioles were sampled, placed in a sealed plastic bag lined with wet paper towels and transported immediately to the laboratory at the Department of Botany in Innsbruck. Samples were stored at + 4 °C until measured within the next one to five days. Fv/Fm values were measured shortly before the measurements using an IMAGING-PAM (M-series with CCD camera, Walz, Effeltrich, Germany) or Mini Pam (Mini-PAM, Heinz Walz GmbH, Effeltrich, Germany, Supplemental Table 2). Fv/Fm values ranging from 0.6 to 0.8 (Supplemental Table 2) indicated that leaf material was healthy and in a physiologically active state.

### Sample preparation

Leaves were carefully cleaned, surface dried and cut longitudinally. Thereby the midrib was removed in order to ensure homogeneity and comparability across the different species. One half of the leaf was weighed and placed into the measuring cell of a DSC (Microcalvet, Setaram, Caluire-et-Cuire, France). Between 9.40 and 33.46 mg of leaf material were used, with the amount visually estimated based on species-specific leaf tissue differences. The other half of the same leaf was used for measurements of basic chlorophyll fluorescence by use of a chlorophyll fluorometer (PAM101, Heinz Walz, Effeltrich, Germany). During the measurements, the leaf blade was placed under the end of the PAM-fiber optic. In parallel, adjacent leaves or leaf parts were measured by two additional chlorophyll fluorometers (PAM101, Heinz Walz, Effeltrich, Germany). For species with divided leaves, i.e. *Ranunculus glacialis* and *Alchemilla alpina* agg., parts of the same leaflet were used for DSC and PAM101 in parallel and adjacent leaflets parts were used for the other two PAMs. For *Saxifraga exarata*, which had very small leaves, multiple leaves had to be used, but leaves originated from the same rosette. For the classical heat tolerance test, which requires 60–100 leaves, leaves of the same sampling pool (sampled at the same time and location from several individuals) were used.

### Determination of heat thresholds by Differential Scanning Calorimetry

A differential scanning calorimeter (DSC, Microcalvet, Setaram, Caluire-et-Cuire, France) was used to study changes in the heat flow of leaves during controlled heating and to derive parameters indicative of heat damage. Before the leaf sample was placed in the standard cell (Setaram, Caluire-et-Cuire, France), the fresh mass of the leaf sample was determined (Quintix 65-1S, Sartorius, Göttingen, Germany). The sample temperature was increased at a constant rate of 1 °C min^−1^ from 25 °C to 65 °C. After heating and cooling back to 25 °C, the same (heat killed) leaf piece was reheated to 65 °C under identical conditions. Both runs lasted 160 min in total. DSC records the heat flow (mW) as a function of temperature and time. Heat flow was normalized for leaf fresh weight (g) and plotted against sample temperature.

### Heat threshold of PSII using chlorophyll fluorescence

Thermotolerance of photosystem II was determined according to a method that was originally described by Schreiber and Berry [35] and Bilger et al. [2] but adapted as described in Neuner and Pramsohler [29]. Three PAM101 fluorometers were used in parallel (Walz, Effeltrich, Germany). Leaves were placed under the end of the fiber optics that were fixed in a metal block (size 12 × 8 × 6 cm). A copper-constantan thermocouple was attached to the lower leaf side of each leaf in order to measure leaf temperature. The entire metal block containing the leaves, the fibre optics and thermocouples was wrapped in a heat-resistant plastic bag and placed in a thermostatic water bath (AC200, Thermo Fisher Scientific, Newington USA) at a temperature of 25 °C for at least 10 min. The water bath temperature was then increased at a constant rate of 1 °C min^−1^ to 65 °C. Basic chlorophyll fluorescence (F_0_) and leaf temperatures were recorded continuously with a data logger (CR 10, Campbell Scientific Instruments, Logan Utah, USA). From the T-F0 curves, the critical temperature (Tc) and the peak temperature (Tp) for thermotolerance of PSII were determined graphically (Supplemental Figure S1).

### Foliar heat tolerance (LT_50_)

The classical heat test [16] was adapted and the same heat treatment was used as in the other experiments, i.e. leaves were heated from 25 °C to different target temperatures up to 65 °C at a constant heating rate of 1°Cmin^−1^ in a water bath (AC200, Thermo Fisher Scientific, Newington USA). Ten target temperatures were chosen with 2 K difference, depending on the heat tolerance of the species, starting from 38 or 40 up to 54 or 56. In addition, one sample was heated to 65 °C to represent full damage and one control was left unheated to represent no heat damage. Six to ten replicates were tested for each target temperature using leaves from different individuals for each target temperature. Samples were placed on wet filter paper and plunged inside heat-durable and watertight plastic bags into the water bath and heated. After heat treatment, leaf samples were exposed to room temperature under dim light to allow heat damage to develop, which took two up to three days depending on the species. The extent of heat damage was assessed by measuring chlorophyll fluorescence (Fv/Fm value) using an IMAGING-PAM (M-series with CCD camera, Walz, Effeltrich, Germany) or Mini Pam (Mini-PAM, Heinz Walz GmbH, Effeltrich, Germany). Fv/Fm was plotted against target temperatures. A sigmoidal function was fitted to the data to calculate LT (lethal temperature) values as a measure of heat tolerance [Eq. (1)].

1 $$y=A2+\frac{A1-A2}{1+{\left(\frac{x}{x0}\right)}^{p}}$$

y: dependent variable (LT%) x: independent variable (temperature) × 0: temperature describing the inflection point of the curve (LT_50_) A1, A2: minimum and maximum of the curve, p: parameter determining the slope of the curve.

According to a modified protocol in Stegner et al. [39] for each temperature treatment three samples were used to calculate the curves and the procedure was repeated 250 times. The LT_10_ and LT_50_ values indicate the temperature that causes 10 and 50% tissue damage, respectively.

### Data analysis

Data were visualized and analyzed using the software R [33]. Statistical differences in the measured parameters between species were tested using one-way ANOVAs or by generalized linear models (GLMs), with species as a factor and trait as the dependent variable. Model assumptions were tested using diagnostic plots, and homogeneity of variances was tested using Levene’s test. If error variances were unequal, i.e. for LT_10_, LT_50_, T_C_ and T_pII_, a GLM was used instead of ANOVA. To illustrate differences between species, mean differences and their 95% confidence intervals were calculated for each species pair. P-values from pairwise comparisons were adjusted using Tukey’s HSD. The correlation between the parameters measured by the different methods was tested by Pearson rank correlation. To calculate correlation coefficients of DSC and T-F0 parameters, values obtained from the same leaf were used; to calculate correlation of DSC and T-F0 parameters with LT10 and LT50.

## Results

### DSC curve patterns and repeatability across leaves and species

**Fig. 1:**
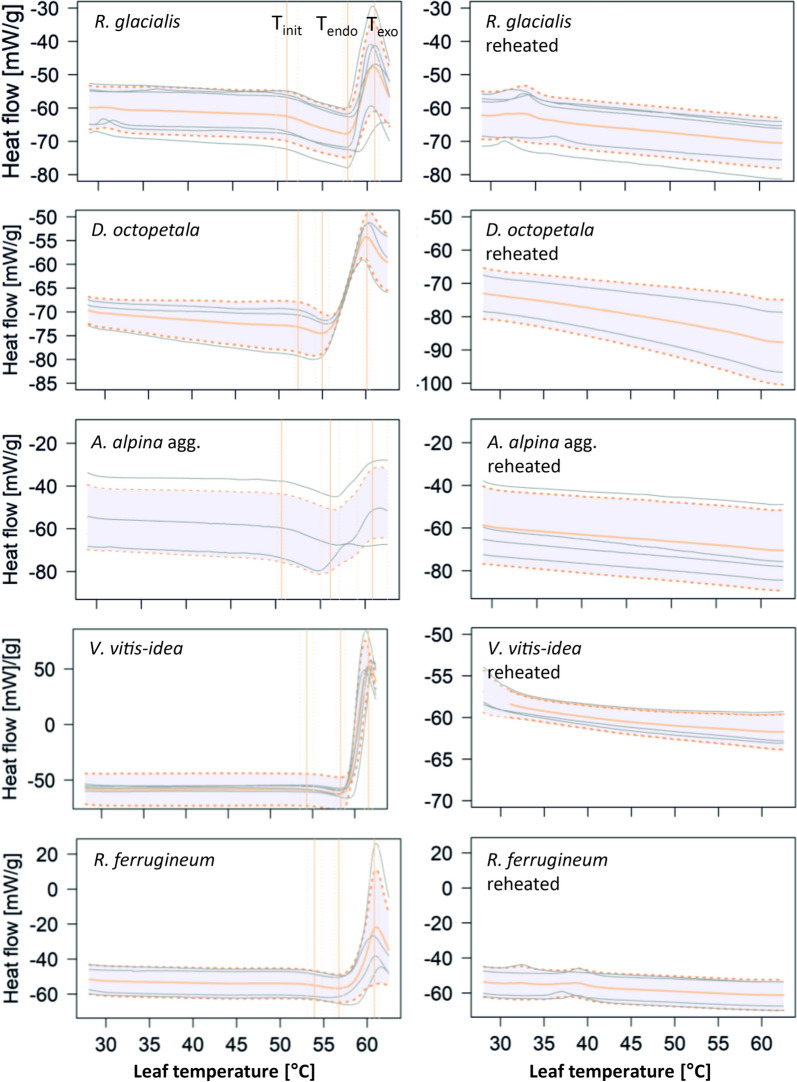
Typical pattern of heat flow (normalized per gram fresh leaf weight) in response to increasing temperature, measured in different species. Grey lines correspond to individual leaves, orange lines to the mean of different leaves of the same species at a particular temperature (rounded to one digit values), dotted orange lines to the mean ± standard deviation of the heat flux. The area between the mean ± standard deviation is shaded in grey. The vertical orange lines correspond to parameters calculated from the curves, i.e. T_init_, T_endo_ and T_exo_. On the left are the response curves of the vital leaves and on the right the response curves of the same leaves reheated after heat killing. Sample size was n = 3 for *Alchemilla alpina* agg. and *Dryas octopetala*, n = 4 for *Rhododendron ferrugineum*, n = 5 for *Ranunculus glacialis* and for *Vaccinium vitis-idea* for the measurement of vital leaves, which were further analysed

Temperature-induced molecular changes were detected using DSC, indicating endo- or exothermic transitions in leaves (Fig. 1). Measurements of heat flow from leaf samples during temperature-controlled heating (1 °C min^−1^) using DSC showed a typical curve pattern that was repeatable across different leaves and species (Fig. 1) and sampling dates during the vegetation period and years (Supplemental Figure S2). In the cold acclimated state (in winter) the curve pattern could be different (data not shown). It was characterized by an initial linear endothermic decrease, followed by a more rapid endothermic decrease and a large exotherm at high temperature (Fig. 1). As key parameters, the peak temperatures of the endotherm (T_endo_) and exotherm (T_exo_) were determined, as well as a point of intersection of two linear equations lower than T_endo_ (T_init_, Fig. 1). The temperature ranges for fitting linear equations, were selected on the basis of their R^2^ individually for each measurement. The first linear was selected by passing a 10 K temperature band through the data (from 29 °C to T_endo_) and selecting the equation with the highest R^2^. The second was defined as being at a higher temperature than the highest value of the first equation obtained and at a lower temperature than T_endo_. A 3 K temperature band was passed through the data and the equation with the highest R^2^ was selected. The intersection point of both linears was then calculated and defined as the parameter T_init._ These parameters are independent of the mass of the sample (which varied) and describe the critical temperatures of the phase transitions within the leaf.

Interestingly, the described curve pattern could only be observed in vital (i.e. physiologically intact, see Supplemental Table 2) leaves before heat treatment. When, after heat killing, the same leaf sample was reheated, samples only showed a successive, rather linear decrease in heat flow without the striking jag and peak of T_endo_ and T_exo_ (Fig. 1).

### Leaf heat tolerance and thermotolerance of PSII

**Fig. 2:**
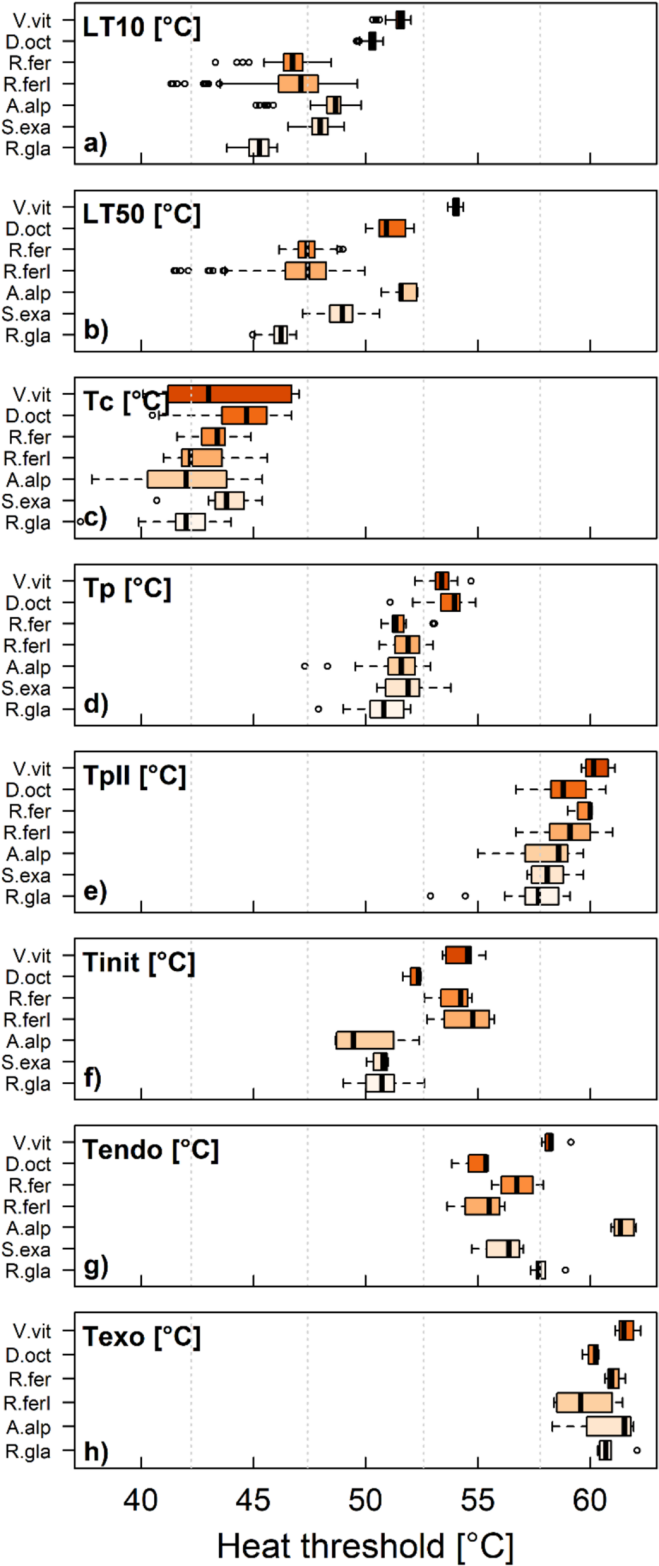
Heat tolerance of different mountain species (*Vaccinium vitis-idea* [V. vit], *Dryas octopetala* [D. oct], *Rhododendron ferrugineum* [R. fer, R. ferI], *Alchemilla alpina* agg. [A. alp], *Saxifraga exarata* [S. exa], *Ranunculus glacialis* [R. gla]), characterized by **a**, **b**) a classical heat test (LT_10_, LT_50_), **c**–**e**) the increase in basic chlorophyll fluorescence (T_c_, T_p_, T_p__II_) and **f**–**h**) DSC (T_init_, T_endo_, T_exo_) in response to heating. Boxplots show the median and the 25/75 percentiles. Whiskers are 1.5 times interquartile ranges, values outside are indicated as outliers. *T_exo_ could not be determined in *S. exarata.* The number of measurements, n, was as follows: for T-F0 measurements, i.e. T_c_ and T_p_ (n = 12 for A. alp, D. oct, and R. fer, n = 14 for V. vit, n = 19 for R. gla, n = 21 for R. ferI and S. exa); for DSC measurements, i.e. T_init_, T_endo_ and T_exo_ (n = 3 for A. alp and D. oct, n = 4 for R. fer and R. ferI, n = 5 for R. gla, V. vit, and S. exa). For the determination of LT_10_ and LT_50_ 6–10 leaves per species and temperature treatment were used and values bootstrapped as described in the methods section

According to their native habitat and sampling date (Supplemental Table S1), the studied species differed in their sensitivity to heat stress, as exemplified by differences in LT_10_ (F_6_ = 1817.7, p < 0.001, Fig. 2, Supplemental Table S3–4), LT_50_ (F_6_ = 3365.6, p < 0.001,, Fig. 2, Supplemental Table S3–4) and parameters determined from T-F0 curves (T_C_: F_6_: 4.1239, p < 0.001; T_p_: F_6_ = 19.967, p < 0.001; T_pII_: F6 = 11.991, p < 0.001, Fig. 2, Supplemental Table S3–4). Correspondingly, the species also differed in the parameters T_init_ (F_6_ = 12.315, p < 0.001, Fig. 2, Supplemental Table S3–4) and T_endo_ (F_6_ = 29.821, p < 0.001, Fig. 2, Supplemental Table S3–4) determined from the DSC curves, but not in T_exo_ (*ns.* F_5_ = 2.2928, p = 0.086, Fig. 2, Supplemental Table S3–4).

### Correlation of obtained parameter

Considering that correlations above 0.5 are considered as informative, T_c_, T_p_ and T_pII_ are correlated with LT_50_ and with among each other. T_pII_ is correlated with T_p_, T_init_ and T_exo_ and with LT_50_. DSC parameters are correlated with various parameters: T_init_ with T_p_ and T_pII_, T_endo_ with T_exo_ and T_exo_ additionally with T_pII_ (Supplemental Table S5). Correlations among species were weak; however, a higher and specific correlation was observed within species (Supplemental Figure S3, Supplemental Table S5–6).

## Discussion

### Heat damage to plant leaves can be detected using DSC

**Fig. 3:**
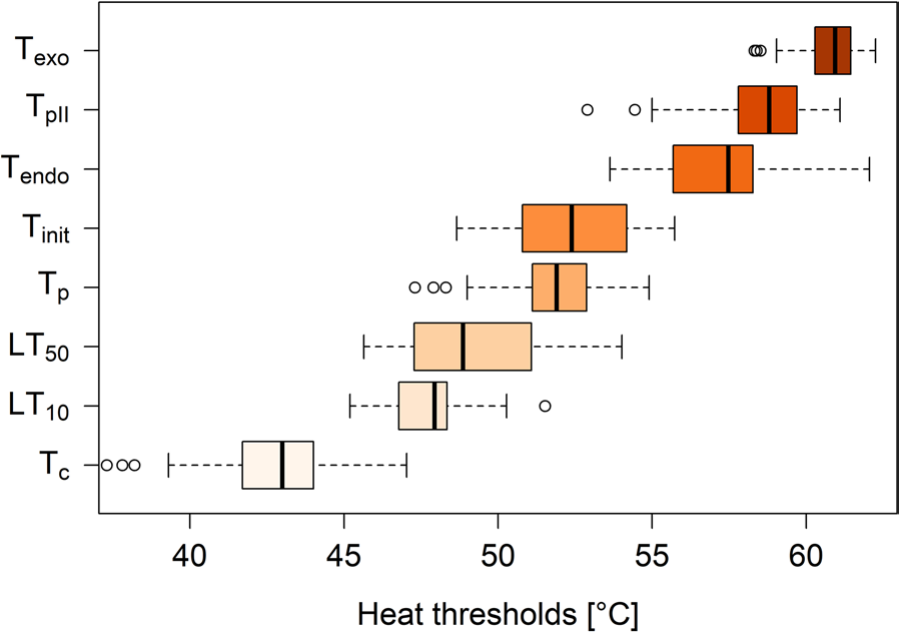
Temperature range of determined parameters, obtained by different methods. Values of all investigated species are pooled. Boxplots show the median and the 25/75 percentiles. Whiskers are 1.5 times interquartile ranges, values outside are indicated as outliers. The number of measurements, n, for each trait in ascending order from T_c_ to T_exo_, was n = 111, n = 50, n = 50, n = 111, n = 29, n = 29, n = 111, n = 24

The observed DSC curve pattern obtained during temperature-controlled heating of leaves was typically divided into three sections and was reproducible across leaves, species, and sampling dates, allowing robust parameters to be derived across measurements (Fig. 1, Supplemental Figure S2). An initial linear, endothermic decrease of heat flow in response to increasing temperature is likely to correspond to the heat capacity of the leaf material as heating of the filled compared to the empty reference cell requires more energy to reach the same target temperature. Temperatures below 40 °C were non-lethal for the leaves studied as confirmed by the simultaneous measurement of LT_50_ and T-F0 parameters (Supplemental Table S3, Figs. 2, 3), which is consistent with the literature on heat tolerance of adult mountain plant leaves during short-term heat exposure [19, 27, 36]. At higher temperatures between 42 and 59 °C, which are normally severely damaging or lethal to most mountain plant leaves (except for *Sempervivum* 64 °C; [27]), heat flow decreased steeply until an endotherm (T_endo_) at around 55–60 °C and a pronounced exotherm (T_exo_) at around 60–61 °C (depending on the species) were reached (Figs. 1, 2 and 3). The detrimental effect of temperatures above 45–50 °C was confirmed by simultaneous measurements of LT_50_ and T_p_ (Supplemental Table S3, Figs. 1, 2 and 3). The temperature range, in which the initial linear decrease decreases steeply until a pronounced endotherm is reached, is likely to represent the accumulation of cellular damage by physicochemical processes and structural changes in leaves in response to heat exposure (e.g. increased membrane fluidity, disintegration of membranes, protein denaturation and agglomeration of cellular compounds, [36]), as the loss of structural order can be associated with the release or consumption of energy. For example, thermal protein denaturation is usually an endothermic process**,** whereas protein aggregation is usually an exothermic process [3, 7, 21, 31]. Evidence that the observed endotherm and exotherm were related to the loss of structural integrity and represent irreversible events was provided by the absence of a pronounced endo- and exotherm in DSC measurements of heat killed leaves, which were reheated again after the initial heat treatment (Fig. 1). Thus, the parameters calculated from the DSC curves are likely to reflect well the effect of high temperature on molecular integrity, in particular the inflection point (T_init_) and the temperature of the endotherm (T_endo_), allowing robust and informative parameters to be obtained.

### Heat damage emerges successively

Heat damage occurs progressively and manifests itself in parameters, indicative for different physiological processes and damaging events (Figs. 2, 3). Above favourable temperatures, the first process responding to increasing temperature was photosynthesis, evidenced by an increase in basic fluorescence (T_c_) that indicates reversible impairment of PSII and was measured at 42.9 ± 2 °C depending on the species and leaf (Figs. 2, 3). This is in agreement with the literature, which reports PSII to be the most heat susceptible subprocess of photosynthesis [46]. PSII is known to be highly heat-labile, which could result from the properties of the thylakoid membrane; dissociation of the oxygen evolution complex causes dissociation of the Mn stabilising protein at the PSII reaction center [47] and eventually of the D1 and D2 proteins. Interestingly, T_c_ shows a large variation between species, but also within species (Figs. 2, 3). At a temperature which is in the middle between the T_c_ and the T_p_ at around 47.5 ± 1.4 °C, the point of no return is reached where heat causes irreversible damage to PSII, which nicely matches with LT_10_ which lies at 48.1 ± 2.2 °C (Figs. 2, 3).

Manifestation of irreversible damage to leaves, evidenced by LT_10_ and LT_50_ was measured at 48.1 ± 2.2 °C and 49.5 ± 2.7 °C respectively (Fig. 3, Supplemental Table S3). In this range and above at around 51.9 ± 1.4 °C, T_p_ indicates irreversible impairment of photosynthesis and T_init_ measured at 52.3 ± 2 °C indicates the onset of denaturation of cellular compounds (Fig. 3, Supplemental Table S3). As LT_50_ detects also damage evolving slower, such as the inactivation of enzymes in chloroplasts and mitochondria an the inhibition of protein synthesis after heat treatment [36], it seems likely that it is found at slightly lower temperatures than T_init_ or T_p_. Compared to other studies LT_50_ lies at comparably higher temperatures. This may be caused by the fact that, however, in the current study LT_50_ was determined by heating the leaves at a rate of 1°C min^−1^ till targets were reached in order to use the same heat treatment as in the other methods, whereas in the classical heat tolerance test samples remain usually exposed at target heat temperatures for 30 min [2, 29]. This illustrates well the dose effect of heat on leaves that was proposed earlier [19, 36], and recently demonstrated by Neuner and Buchner [28] according to the dose–effect law. However, all three parameters (LT_50_, T_p_ and T_init_) seem to indicate detrimental heat effects to leaves quite well and likely provide ecologically informative parameters of heat injury.

Pre-experiments with leaves of *R. glacialis*, sampled at the same date and time and from the same individuals, as was used for the described experiments, indicate that membrane leakage occurs after impairment of photosynthesis (T_c_) clearly before T_endo_ and T_exo_ (data not shown). T_endo_ and T_exo_ the most prominent peaks in the DSC curves (Fig. 1), likely indicate denaturation and aggregation of proteins and cellular compounds, respectively [7] and lie at relatively high temperatures at 57.5 ± 2.2 °C and 60.7 ± 1.1 °C respectively. Thus, damage to membranes may occur before proteins and other cellular compounds are denatured. T_endo_ may therefore be interpreted as heavily damaging or lethal temperature connected to protein denaturation; at T_exo_ a lethal temperature is reached. As T_exo_ does not differ between the species of different habitats (Fig. 2) and rather represents a lethal point of complete aggregation of cellular material it seems to be rather uninformative in an ecological context. However, the protective effect of heat shock proteins (HSPs) expression, which is a well described response under heat shock and a basic mechanism of thermotolerance, results from the maintenance of protein homeostasis by preventing the aggregation of heat-denatured proteins and facilitation of their refolding [9]. Interestingly, T_pII_ a parameter observed in former studies using T-F0 curves [29] but hardly ever interpreted lies between T_endo_ and T_exo_ at 58.7 ± 1.4 °C and roughly matches T_endo_. Interestingly, in the winter state, the typical first peak in the T-F0 curve was missing (pre-experiments with *Pinus mugo* and *R. ferrugineum*), which was also reported in Neuner & Pramsohler [29]. The lack of T_p_ coincides, with a severe reduction of T_exo_, that is hardly detectable in the winter state (data not shown). Thus, it seems likely that the typical pattern observed by the DSC method is related to PSII.

A relatively weak correlation (Supplemental Table S5, Supplemental Figure S3) between the parameters obtained by different methods, when all different species are pooled may indicate that processes leading to heat injury emerge at different rates or in a different way in different species. The thermal thresholds potentially depend on the structure and composition of their leaves, cells and membranes (e.g. specific lipid pattern in biomembranes, the abundance of chaperones, and heat shock proteins, Schulze et al. [36]). In order to test whether or not we can detect heat injury by DSC, we chose species that differ significantly in their ecological niche and thermal adaptation (Supplemental Table S1)—thus difference in their response to heat exposure were likely (not only in the temperature but also in the way how heat damage evolves as well as in their thermal history which affect their cellular composition). Congruently, a species-specific relationship between the measured parameters can be observed in the plot (Supplemental Figure S3) and manifests itself in comparable high correlation coefficients when parameters are correlated within a species (Supplemental Table S6). In agreement with the relatively weak correlation when values from species are pooled (Supplemental Table S5), rather weak correlations and species dependent scatter were also observed in former studies correlating T-F0 parameters with LT_50_ [2, 29, 45].

### On the value of the new method

Measuring heat tolerance by DSC allows heat tolerance—or more precisely the temperature at which heat damage occurs—to be determined precisely, easily, and quickly (e.g. within ca. 100 min), and requires only a small amount of material. This is a methodological advantage over the classical heat test, which reflects heat tolerance reasonably well, but is time-consuming, laborious and requires a lot of plant material (e.g. 60 to 100 leaves or leaf discs), which can be critical when leaf material is scarce or the plant species is endangered. Furthermore, the determination of LT_50_ by the classical heat tolerance test depends on the mode of viability assessment and the time interval between heat treatment and viability assessment (see [26]. Compared with the DSC method, which is good at detecting heat injury to a specific leaf (area), it only allows an average heat tolerance value to be obtained from a number of leaves and individuals, depending on the question of advantage or disadvantage. However, the classical heat tolerance test is the only method that can detect heat injury that does not appear immediately in response to temperature treatment.

DSC appears to be most useful for detecting irreversible damage to leaves by structural disruption of membranes and proteins, rather than reversible or transient functional cell impairments, and can therefore complement the T-F0 method and the classical heat test well. In contrast, the T-F0 technique, which is also fast and easy to use, shows the increase in chlorophyll fluorescence of a leaf due to changes in the functionality of PSII, and has its strength in detecting reversible reductions in photosynthesis. Due to a large variation in T_c_, interpreting the thermotolerance of a species at an ecological level using T_c_ only, appears to be difficult (Fig. 2). T-F0 and the classical heat tolerance test provide critical thresholds for heat injury, but these can differ widely by several °C [2, 29]. However, measurements by different methods and the determination of different parameters may be particularly valuable in the study of heat tolerance, as heat damage involves several structures and processes and the sequence appears to differ in different species.

Overall, the new method makes it possible to obtain parameters for comparing critical heat thresholds for species or leaves, which could be used in future research on plant heat tolerance. Novel ways of measuring heat tolerance are urgently needed in the face of ongoing rapid climate change, as physiological thermal limits (in relation to leaf temperatures in present and future) are not known for a wide range of plant species. In addition, rapid and easy methods will facilitate multiple measurements of heat tolerance throughout the season, which is important because heat tolerance is highly plastic and adapted to the current temperature conditions.

## Conclusions

- The results show that the DSC curve patterns are reproducible and provide robust parameters that allow the thermal tolerance of plant leaves to be precisely determined. In particular, the identification of critical temperature limits, at which irreversible damage to molecular integrity occurs, is of great importance for understanding the adaptation mechanisms of plants to increasing temperatures.
- The analysis shows that heat damage occurs gradually and manifests itself in various physiological processes. The method offers a clear advantage over traditional tests, as it requires less material and can be carried out more quickly. This is particularly relevant in the light of climate change, which is challenging the thermal limits of many plant species.
- Simultaneous measurements of parameters by different methods indicative of different processes occurring in response to heat stress, such as reversible and irreversible impairment of photosynthesis, denaturation of cellular compounds, membrane damage and whole tissue damage, allow a better understanding of the evolution of heat damage in leaves. Due to a species-specific sequence in the damage process, the ecological characterization of thermal thresholds benefits from the consideration of multiple parameters.
- To summarize, the DSC method not only represents a valuable addition to existing methods, but also opens up new perspectives for research into the heat tolerance of plants. Given the urgency to understand the effects of climate change, the development and application of such innovative methods is crucial to ultimately understand the adaptability of plants to changing environmental conditions.

## Supplementary Information

Supplementary Material 1.

## Data Availability

No datasets were generated or analysed during the current study.

## Abbreviations

LT_10_: Values indicate the temperature that causes 10% tissue damage. It is calculated from a sigmoidal equation fitted to values of tissue damage in response to exposure temperature.
LT_50_: Values indicate the temperature that causes 50% tissue damage. It is calculated from a sigmoidal equation fitted to values of tissue damage in response to exposure temperature.
T_c_: Values indicate the temperature at which the basic fluorescence in the dark-adapted state of a leaf starts to rise. It is an indicator of heat-induced impairments to PSII.
T_p_: Values indicate the temperature at which the basic fluorescence in the dark-adapted state of a leaf has its first peak and is an indicator of irreversible damage to PSII.
T_pII_: Values indicate the temperature at which the basic fluorescence in the dark-adapted state of a leaf has its second peak and is an indicator of irreversible damage to PSII.
T_init_: Values indicate the temperature at which the heat flow of a leaf starts to decrease non-linearly when measured in a DSC.
T_endo_: Values indicate the temperature at which the heat flow of a leaf shows a pronounced endotherm when measured in a DSC.
T_exo_: Values indicate the temperature at which the heat flow of a leaf shows a pronounced exotherm when measured in a DSC

## References

[1] Aitken SN, Yeaman S, Holliday JA, Wang T, Curtis-McLane S. Adaptation, migration or extirpation: climate change outcomes for tree populations. Evol Appl. 2008;1:95–111. 25567494 10.1111/j.1752-4571.2007.00013.x PMC3352395

[2] Bilger H-W, Schreiber U, Lange O. Determination of leaf heat resistance: comparative investigation of chlorophyll fluorescence changes and tissue necrosis methods. Oecologia. 1984;63:256–62. 28311022 10.1007/BF00379886

[3] Briones-Martínez R, Juárez-Juárez M, Oliver-Salvador M, Cortés-Vázquez M. Influence of enzymatic hydrolysis on functionality of plant proteins: DSC studies. J Therm Anal Calorim. 1997;49:831–7.

[4] Corlett RT, Westcott DA. Will plant movements keep up with climate change? Trend Ecol Evol. 2013;28:482–8. 10.1016/j.tree.2013.04.003 23721732

[5] Cramer WA, Whitmarsh J, Low PS. Differential scanning calorimetry of chloroplast membranes: identification of an endothermic transition associated with the water-splitting complex of photosystem II. Biochemistry. 1981;20:157–62. 7470468 10.1021/bi00504a026

[6] Donovan J, Mapes C. Multiple phase transitions of starches and Nägeli amylodextrins. Starch. 1980;32:190–3.

[7] Fitzsimons SM, Mulvihill DM, Morris ER. Denaturation and aggregation processes in thermal gelation of whey proteins resolved by differential scanning calorimetry. Food Hydrocoll. 2007;21:638–44.

[8] Freire E. Differential scanning calorimetry. Protein stability and folding: theory and practice. Method Mol Biol. 1995;40:191–218. 10.1385/0-89603-301-5:191 7633523

[9] Haslbeck M, Vierling E. A first line of stress defense: small heat shock proteins and their function in protein homeostasis. J Mol Biol. 2015;427:1537–48. 25681016 10.1016/j.jmb.2015.02.002 PMC4360138

[10] Calvin K, Dasgupta D, Krinner G, Mukherji A, Thorne PW, Trisos C, Romero J, Aldunce P, Barrett K, Blanco G, Cheung WW. IPCC, 2023: climate change 2023: synthesis report contribution of working groups I, II and III to the sixth assessment report of the intergovernmental panel on climate change [Core Writing Team, Lee H. and Romero J. (Eds.)]. Geneva: IPCC; 2023. p. 35–115.

[11] Jezkova T, Wiens JJ. Rates of change in climatic niches in plant and animal populations are much slower than projected climate change. Proceed R Soc B Biol Sci. 2016;283:20162104. 10.1098/rspb.2016.2104 PMC5136599 27881748

[12] Johnson CM. Differential scanning calorimetry as a tool for protein folding and stability. Arch Biochem Biophys. 2013;531:100–9. 10.1016/j.abb.2012.09.008. 23022410 10.1016/j.abb.2012.09.008

[13] Jump AS, Peñuelas J. Running to stand still: adaptation and the response of plants to rapid climate change. Ecol Lett. 2005;8:1010–20. 34517682 10.1111/j.1461-0248.2005.00796.x

[14] Kõiv A, Kinnunen PK. Binding of DNA to liposomes containing different derivatives of sphingosine. Chem Phys Lipid. 1994;72:77–86. 10.1016/0009-3084(94)90018-3 7923481

[15] Körner C. Alpine plant life: functional plant ecology of high mountain ecosystems. 3rd ed. Berlin/Heidelberg: Springer Nature; 2021.

[16] Kreeb KH. Methoden zur pflanzenoekologie und bioindikation. Stuttgart: Fischer; 1990.

[17] Krumova S, Todinova S, Dobrikova A, Taneva S. Differential scanning calorimetry of photosynthetic membranes: resolving contributions of major photosynthetic complexes to the thermal transitions. Trend Photochem Photobiol. 2010;12:37–51.

[18] Laczkó-Dobos H, Todinova SJ, Sözer Ö, Komenda J, Kis M, Sallai A, Dobrikova AG, Ughy B, Debreczeny M, Gombos Z. Identification of thylakoid membrane thermal transitions in *Synechocystis sp.* PCC6803 photosynthetic mutants. Photosynth Res. 2011;107:237–46. 21298342 10.1007/s11120-011-9627-3

[19] Larcher W. Physiological plant ecology: ecophysiology and stress physiology of functional groups. Cham: Springer Science & Business Media; 2003.

[20] Lewis RN, Mannock DA, McElhaney RN. Differential scanning calorimetry in the study of lipid phase transitions in model and biological membranes: practical considerations. Method Membr Lipids. 2007;400:171–95. 10.1007/978-1-59745-519-0_12 17951734

[21] Libouga DG, Aguié-Béghin V, Douillard R. Thermal denaturation and gelation of rubisco: effects of pH and ions. Int J Biol Macromol. 1996;19:271–7. 9024903 10.1016/s0141-8130(96)01137-3

[22] Lin L-S, Yuen HK, Varner JE. Differential scanning calorimetry of plant cell walls. Proc Natl Acad Sci. 1991;88:2241–3. 11607163 10.1073/pnas.88.6.2241 PMC51206

[23] Lopez MM, Makhatadze GI. Differential scanning calorimetry, calcium-binding protein protocols. Method Techniques. 2002;2:113–9. 10.1385/1-59259-184-1:113 11859754

[24] Lorenz R, Stalhandske Z, Fischer EM. Detection of a climate change signal in extreme heat, heat stress, and cold in Europe from observations. Geophys Res Lett. 2019;46:8363–74.

[25] Makhatadze GI, Privalov PL. Energetics of protein structure. Adv Protein Chem. 1995;47:307–425. 8561051 10.1016/s0065-3233(08)60548-3

[26] Neuner G, Buchner O. Assessment of foliar frost damage: a comparison of *in vivo* chlorophyll fluorescence with other viability tests. Angew Bot. 1999;73:50–4.

[27] Neuner G, Buchner O. Dynamics of tissue heat tolerance and thermotolerance of PS II in alpine plants. In: Lütz C, editor. Plants in alpine regions. Cham: Springer; 2012.

[28] Neuner G, Buchner O. The dose makes the poison: the longer the heat lasts, the lower the temperature for functional impairment and damage. Environ Exp Bot. 2023;212: 105395.

[29] Neuner G, Pramsohler M. Freezing and high temperature thresholds of photosystem 2 compared to ice nucleation, frost and heat damage in evergreen subalpine plants. Physiol Plant. 2006;126:196–204.

[30] Perkins-Kirkpatrick SE, Lewis SC. Increasing trends in regional heatwaves. Nat Commun. 2020. 10.1038/s41467-020-16970-7. 32620857 10.1038/s41467-020-16970-7 PMC7334217

[31] Privalov PL, Potekhin SA. Scanning microcalorimetry in studying temperature-induced changes in proteins. Method Enzymol. 1986;131:4–51. 10.1016/0076-6879(86)31033-4 3773768

[32] Ralser M, Stegner M, Neuner G. When water turns to ice: control of ice volume and freezing dynamics as important aspects of cold acclimation. Environ Exp Bot. 2024;227: 105957.

[33] R Core Team. _R: a language and environment for statistical computing. R foundation for statistical computing, Vienna, Austria. 2024. https://www.R-project.org/.

[34] Savchenko G, Klyuchareva E, Abramchik L, Serdyuchenko E. Effect of periodic heat shock on the inner membrane system of etioplasts. Russ J Plant Physiol. 2002;49:349–59.

[35] Schreiber U, Berry JA. Heat-induced changes of chlorophyll fluorescence in intact leaves correlated with damage of the photosynthetic apparatus. Planta. 1977;136:233–8. 24420396 10.1007/BF00385990

[36] Schulze ED, Beck E, Buchmann N, Clemens S, Müller-Hohenstein K, Scherer-Lorenzen M. Plant ecology. Cham: Springer; 2019.

[37] Singer SJ, Nicolson GL. The fluid mosaic model of the structure of cell membranes: cell membranes are viewed as two-dimensional solutions of oriented globular proteins and lipids. Science. 1972;175:720–31. 4333397 10.1126/science.175.4023.720

[38] Spink CH. Differential scanning calorimetry. Method Cell Biol. 2008;84:115–41. 10.1016/S0091-679X(07)84005-2 17964930

[39] Stegner M, Lackner B, Schäfernolte T, Buchner O, Xiao N, Gierlinger N, Holzinger A, Neuner G. Winter nights during summer time: stress physiological response to ice and the facilitation of freezing cytorrhysis by elastic cell wall components in the leaves of a nival species. International J Mol Sci. 2020;21(19):7042. 10.3390/ijms21197042 PMC7582304 32987913

[40] Stegner M, Strasser AL, Neuner G. Supercooling cells of frost hardy palm leaves: quantified percentage of frozen water and displacement from thermodynamic equilibrium. Environ Exp Bot. 2024;226: 105895.

[41] Sturtevant JM. Biochemical applications of differential scanning calorimetry. Annu Rev Phys Chem. 1987;38:463–88.

[42] Wahid A, Gelani S, Ashraf M, Foolad MR. Heat tolerance in plants: an overview. Environ Exp Bot. 2007;61:199–223.

[43] Weis E, Berry JA. Plants and high temperature stress. Symp Soc Exp Biol. 1988;42:329–46. 3077863

[44] Winter K, Garcia M, Virgo A. Heat-induced F0-fluorescence rise is not an indicator of severe tissue necrosis in thermotolerance assays of young and mature leaves of a tropical tree species, *Calophyllum inophyllum*. Photosynthetica. 2024;63:46–50. 10.32615/ps.2025.004 PMC12012419 40270906

[45] Winter K, Garcia M, Virgo A (2025). Heat-induced F0-fluorescence rise is not an indicator of severe tissue necrosis in thermotolerance assays of young and mature leaves of a tropical tree species, Calophyllum inophyllum. Photosynth 63(1):46. 10.32615/ps.2025.004 PMC12012419 40270906

[46] Wise RR, Olson, AJ, Schrader SM, Sharkey TD (2004). Electron transport is the functional limitation of photosynthesis in field‐grown Pima cotton plants at high temperature. Plant Cell Environ. 27(6):717–724.

[47] Yamane Y, Kashino Y, Koike H, Satoh K (1998). Effects of high temperatures on the photosynthetic systems in spinach: oxygen-evolving activities, fluorescence characteristics and the denaturation process. Photosynth Res 57:51–59.

[48] Yu L, Christie G. Measurement of starch thermal transitions using differential scanning calorimetry. Carbohyd Polym. 2001;46:179–84.

