# Supplementary material for: A novel method for measuring heat injury in leaves provides insights into the sequence of processes of heat injury development
Source: Plant Methods. 2025 Jul 1;21:89. doi: 10.1186/s13007-025-01404-6 (PMC12210732; doi:10.1186/s13007-025-01404-6)
Supplement: Supplementary file 1 — Supplementary Material 1. [file 13007_2025_1404_MOESM1_ESM.docx]

**Supporting information**

The following Supporting Information is available for this article:

**Supplemental Figure S1.** Typical curve pattern of basic chlorophyll fluorescence in response to increasing temperature and determination of T-F0 parameters.

**Supplemental Figure S2.** Repeatability of the DSC curve pattern in different years.

**Supplemental Figure S3.** Correlation of DSC parameter with T-F0 parameters.

**Supplemental Table S1.** Information about the study species and sampling.

**Supplemental Table S2.** Mean and standard deviation of Fv/Fm of leaves used for the experiments.

**Supplemental Table S3.** Mean and standard deviation of parameters indicative for critical heat thresholds determined by three different methods.

**Supplemental Table S4.** Mean differences, standard error, 95% confidence intervals, t ratios and p values of the differences between species for each parameter.

**Supplemental Table S5.** Pearson correlation coefficients of the measured parameters.

**Supplemental Table S6.** Species specific pearson correlation of the T-F0 and DSC parameters.

**Supplemental Figures**


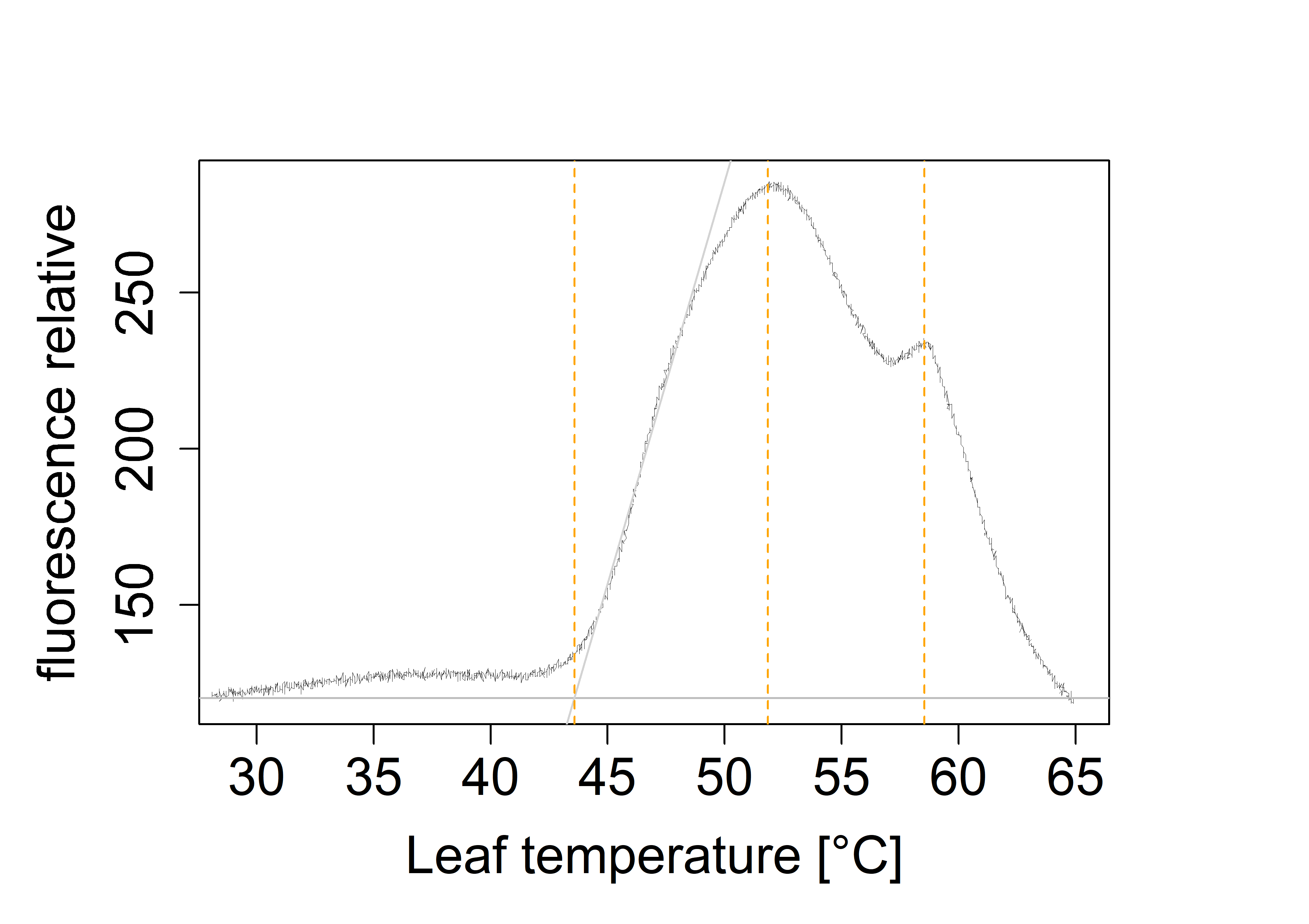


**Supplemental Figure 1. Typical curve pattern of basic chlorophyll fluorescence in response to increasing temperature and determination of T-F0 parameters** at the example of one leaf of *Ranunculus glacialis*. Orange lines correspond to the parameters T_c_, T_p_ and T_pII_ shown in ascending order. Grey lines correspond to two linear equations fitted to the data in order to determine T_c_.


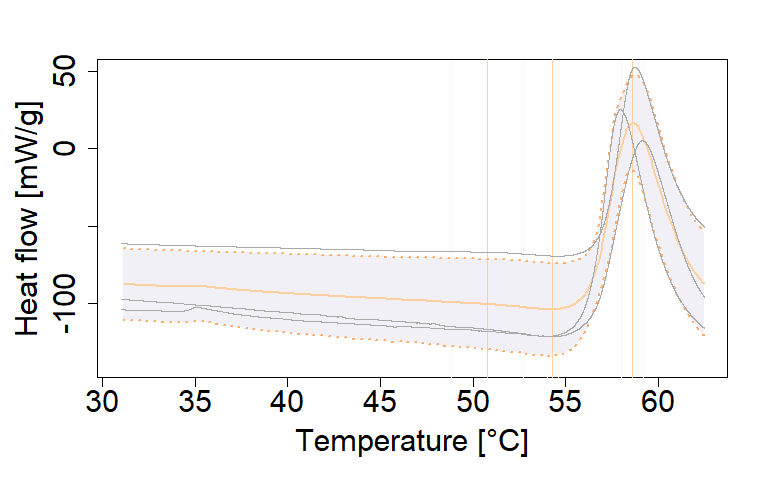


**T_exo_**

**T_endo_**

**T_init_**

**Supplemental figure 2. Repeatability in different years.** Typical pattern of heat flow (normalized per gram leaf fresh weight) in response to increasing temperature, measured on leaves of *R. ferrugineum* in June 2023. Grey lines correspond to individual leaves, orange lines to the mean of different leaves at a particular temperature (rounded to one digit values), dotted orange lines to the mean +/- standard deviation of the heat flux. The area between the mean +/- standard deviation is shaded in grey. The vertical orange lines correspond to parameters calculated from the curves, i.e. T_init_, T_endo_ and T_exo_. Sample size, n=3.


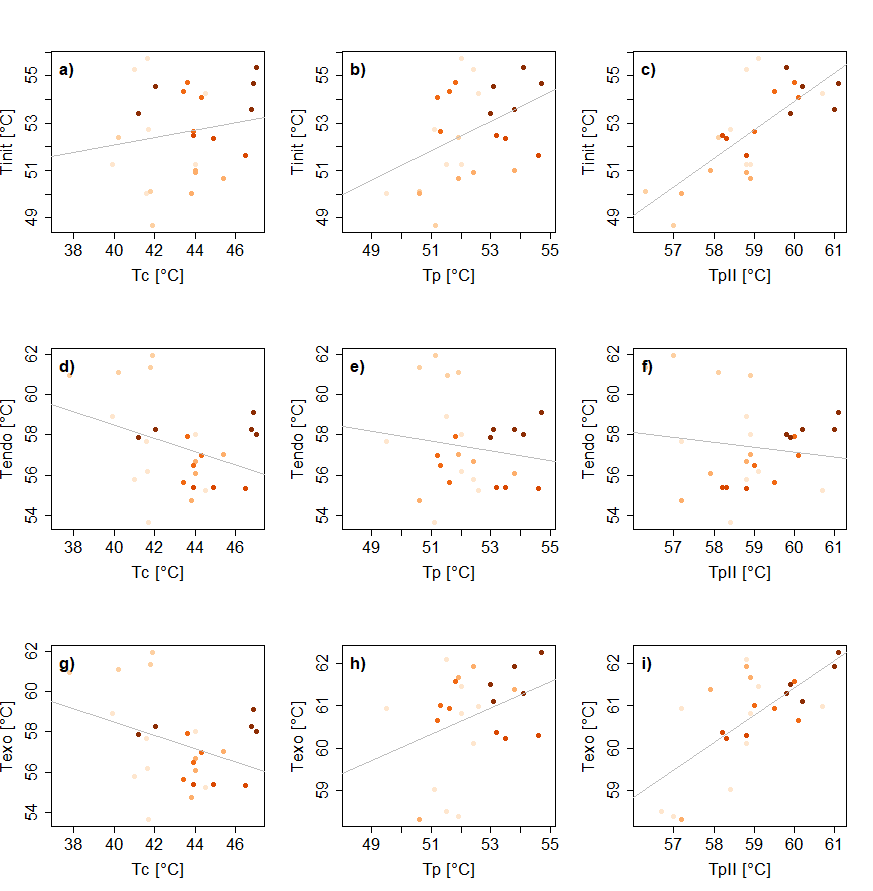


**Supplemental Figure 3.** Correlation of DSC parameter with T-F0 parameters. Colors correspond to values measured from the same species at the same date.

**Supplemental Tables**

**Supplemental Table S1.** The selected alpine study species, family and native habitat, sampling date and site.

| Species | Native habitat | Sampling date | Sampling site |
| --- | --- | --- | --- |
| *Alchemilla alpina* agg.  (Rosaceae) | Subalpine-alpine meadows | 15.5.2024 | Innsbruck, Botanical Garden, 608m, 47,267940°N 11,380608°E |
| *Dryas octopetala*  (Rosaceae) | subalpine-alpine calcerous rocky sites, stony grasslands | 6.6.2024 | Innsbruck, Botanical Garden, 608m, 47,267940°N 11,380608°E |
| *Ranunculus glacialis*  (Ranunculaceae) | subnivale siliceous rocky sites, moraines | 15.07.2024 | Schaufeljoch, 2805m 46,989167 °N 11,116389 °E |
| *Rhododendron ferrugineum*  (Ericaceae) | subalpine forests, pastures, dwarf shrubs on silicate | 21.6.2023  3.7.2024  11.7.2024 | Alpine Garden Patscherkofel, 1950m, 47,210711°N 11,451343°E |
| *Saxifraga exarata*  (Saxifragaceae) | subalpine-alpine siliceous rocky sites | 15.07.2024 | Schaufeljoch, 2805m 46,989167 °N 11,116389 °E |
| *Vaccinium vitis-idea*  (Ericaceae) | subalpine dwarf shrubs, mountain forests, moors | 2.6.2024 | Obernberg am Brenner, 1746m, 46,988766°N 11,393416°E |

**Supplemental Table 2. Mean and standard deviation of Fv/Fm** measured on leaves shortly before the described experiments. Fv/Fm values were measured on a subset of the investigated leaves, e.g. 50-70 leaves per species.

| Species | Fv/Fm |
| --- | --- |
| *Alchemilla alpina* agg. | 0.82 ± 0.04 |
| *Dryas octopetala* | 0.79 ± 0.06 |
| *Ranunculus glacialis* | 0.69 ± 0.03 |
| *Rhododendron ferrugineum* | 0.71 ± 0.05 |
| *Rhododendron ferrugineum I* | 0.70 ± 0.03 |
| *Saxifraga exarata* | 0.59 ± 0.04 |
| *Vaccinium vitis-idaea* | 0.78 ± 0.03 |

**Supplemental Table 3. Critical heat thresholds (°C) determined by three different methods:** Mean and standard deviation of different parameters calculated from measurements of heat dependent leaf tissue damage (LT_10_, LT_50_), the change in basic chlorophyll fluorescence (T_c_ ,T_p_, T_pII_) and by heat flow obtained by DSC (T_init_, T_endo_, T_exo_). *T_exo_ could not be determined in *S. exarata.*

| Species | LT_10_ | LT_50_ | T_c_ | T_p_ | T_pII_ | T_init_ | T_endo_ | T_exo_ |
| --- | --- | --- | --- | --- | --- | --- | --- | --- |
| *Alchemilla alpina* agg. | 48.3 ± 1.0 | 51.7 ± 0.5 | 43.8 ± 1.3 | 51.9 ± 1.1 | 58.2 ± 1.1 | 50.7 ± 0.4 | 56.1 ± 1.0 | 60.8 ± 1.7 |
| *Dryas octopetala* | 50.3 ± 0.2 | 51.1 ± 0.6 | 44.3 ± 2.0 | 53.6 ± 1.1 | 59.0 ± 1.1 | 52.2 ± 0.4 | 55.0 ± 0.8 | 60.1 ± 0.3 |
| *Ranunculus glacialis* | 44.9 ± 0.6 | 46.2 ± 0.4 | 41.6 ± 2.2 | 50.7 ± 1.1 | 57.5 ± 1.1 | 50.7 ± 1.3 | 57.9 ± 0.6 | 60.9 ± 0.6 |
| *Rhododendron ferrugineum* | 46.8 ± 1.5 | 47.4 ± 1.6 | 42.8 ± 1.3 | 51.9 ± 0.7 | 59.0 ± 0.7 | 54.5 ± 1. 3 | 55.2 ± 1.1 | 59.8 ± 1.3 |
| *Rhododendron ferrugineum I* | 46.9 ± 0.6 | 47.2 ± 0.6 | 43.3 ± 0.9 | 51.6 ± 0.7 | 59.8 ± 0.7 | 54.0 ± 0.9 | 56.7 ± 1.0 | 61.0 ± 0.4 |
| *Saxifraga exarata* | 47.9 ± 0.5 | 48.9 ± 0.8 | 42.1 ± 2.2 | 51.3 ± 1.4 | 58.2 ± 1. 4 | 50.0 ± 1.7 | 61.5 ± 0.5 | na* |
| *Vaccinium vitis-idaea* | 51.5 ± 0.3 | 54.0 ± 0.2 | 43.6 ± 2.5 | 53.4 ± 0.6 | 60.2 ± 0.6 | 54.3 ± 0.8 | 58.3 ± 0.5 | 61.6 ± 0.5 |
|  |  |  |  |  |  |  |  |  |

**Supplemental Table 4. Mean differences, standard error, 95% confidence intervals, t ratios and p values of the differences between species for each parameter.** p-values were adjusted by Tukeys HSD. p-values < 0.5 are given in bold.

| Comparison | Mean difference | SE | Lower CI | Upper CI | t ratio | p value |
| --- | --- | --- | --- | --- | --- | --- |
|  | **T_c_** | | | | | |
| *A. alpina* agg. – *D. octopetala* | -0.49 | 0.77 | -2.80 | 1.82 | -0.64 | 1.00 |
| *A. alpina* agg. – *R. ferrugineum* | 0.52 | 0.77 | -1.80 | 2.83 | 0.67 | 0.99 |
| *A. alpina* agg. – *R. ferrugineum I* | 1.03 | 0.68 | -1.02 | 3.08 | 1.51 | 0.74 |
| *A. alpina* agg. – *R. glacialis* | 2.18 | 0.69 | 0.10 | 4.27 | 3.14 | **0.03** |
| *A. alpina* agg. – *S. exarata* | 1.70 | 0.68 | -0.35 | 3.75 | 2.50 | 0.17 |
| *A. alpina* agg. – *V. vitis-idea* | 0.15 | 0.74 | -2.08 | 2.38 | 0.20 | 1.00 |
| *D. octopetala – R. ferrugineum* | 1.01 | 0.77 | -1.30 | 3.32 | 1.31 | 0.85 |
| *D. octopetala – R. ferrugineum I* | 1.52 | 0.68 | -0.53 | 3.57 | 2.23 | 0.29 |
| *D. octopetala – R. glacialis* | 2.68 | 0.69 | 0.59 | 4.76 | 3.85 | **0.00** |
| *D. octopetala – S. exarata* | 2.19 | 0.68 | 0.14 | 4.24 | 3.22 | **0.03** |
| *D. octopetala – V. vitis-idaea* | 0.64 | 0.74 | -1.59 | 2.87 | 0.86 | 0.98 |
| *R. ferrugineum – R. ferrugineumI* | 0.51 | 0.68 | -1.53 | 2.56 | 0.76 | 0.99 |
| *R. ferrugineum – R. glacialis* | 1.67 | 0.69 | -0.42 | 3.75 | 2.40 | 0.21 |
| *R. ferrugineum – S. exarata* | 1.18 | 0.68 | -0.86 | 3.23 | 1.74 | 0.59 |
| *R. ferrugineum – V. vitis-idaea* | -0.37 | 0.74 | -2.60 | 1.86 | -0.50 | 1.00 |
| *R. ferrugineum I – R. glacialis* | 1.15 | 0.60 | -0.64 | 2.95 | 1.93 | 0.46 |
| *R. ferrugineum – S. exarata* | 0.67 | 0.58 | -1.08 | 2.42 | 1.15 | 0.91 |
| *R. ferrugineum – V. vitis-idaea* | -0.88 | 0.65 | -2.84 | 1.07 | -1.36 | 0.82 |
| *R. glacialis – S. exarata* | -0.48 | 0.60 | -2.28 | 1.31 | -0.81 | 0.98 |
| *R. glacialis – V. vitis-idaea* | -2.04 | 0.66 | -4.03 | -0.04 | -3.07 | **0.04** |
| *S. exarata – V. vitis-idaea* | -1.55 | 0.65 | -3.51 | 0.40 | -2.39 | 0.21 |
|  |  |  |  |  |  |  |
| Comparison | **Mean difference** | **SE** | **Lower CI** | **Upper CI** | **t ratio** | **p value** |
|  | **T_p_** | | | | | |
| *A. alpina* agg. – *D. octopetala* | -1.70 | 0.42 | -2.96 | -0.44 | -4.05 | **0.00** |
| *A. alpina* agg. – *R. ferrugineum* | 0.34 | 0.42 | -0.92 | 1.60 | 0.81 | 0.98 |
| *A. alpina* agg. – *R. ferrugineum I* | 0.03 | 0.37 | -1.09 | 1.15 | 0.07 | 1.00 |
| *A. alpina* agg. – *R. glacialis* | 1.19 | 0.37 | 0.08 | 2.30 | 3.23 | **0.03** |
| *A. alpina* agg. – *S. exarata* | 0.62 | 0.37 | -0.50 | 1.74 | 1.68 | 0.63 |
| *A. alpina* agg. – *V. vitis-idea* | -1.45 | 0.40 | -2.66 | -0.23 | -3.58 | **0.01** |
| *D. octopetala – R. ferrugineum* | 2.04 | 0.42 | 0.78 | 3.30 | 4.85 | **0.00** |
| *D. octopetala – R. ferrugineum I* | 1.73 | 0.37 | 0.61 | 2.85 | 4.64 | **0.00** |
| *D. octopetala – R. glacialis* | 2.89 | 0.37 | 1.78 | 4.00 | 7.84 | **0.00** |
| *D. octopetala – S. exarata* | 2.32 | 0.37 | 1.20 | 3.44 | 6.24 | **0.00** |
| *D. octopetala – V. vitis-idaea* | 0.25 | 0.40 | -0.96 | 1.47 | 0.62 | 1.00 |
| *R. ferrugineum – R. ferrugineumI* | -0.31 | 0.37 | -1.43 | 0.81 | -0.84 | 0.98 |
| *R. ferrugineum – R. glacialis* | 0.85 | 0.37 | -0.26 | 1.96 | 2.31 | 0.25 |
| *R. ferrugineum – S. exarata* | 0.28 | 0.37 | -0.83 | 1.40 | 0.76 | 0.99 |
| *R. ferrugineum – V. vitis-idaea* | -1.79 | 0.40 | -3.00 | -0.57 | -4.41 | **0.00** |
| *R. ferrugineum I – R. glacialis* | 1.17 | 0.31 | 0.22 | 2.11 | 3.71 | **0.01** |
| *R. ferrugineum – S. exarata* | 0.60 | 0.32 | -0.36 | 1.55 | 1.88 | 0.50 |
| *R. ferrugineum – V. vitis-idaea* | -1.48 | 0.35 | -2.54 | -0.41 | -4.16 | **0.00** |
| *R. glacialis – S. exarata* | -0.57 | 0.31 | -1.51 | 0.37 | -1.81 | 0.54 |
| *R. glacialis – V. vitis-idaea* | -2.64 | 0.35 | -3.70 | -1.58 | -7.51 | **0.00** |
| *S. exarata – V. vitis-idaea* | -2.07 | 0.35 | -3.14 | -1.00 | -5.84 | **0.00** |
| Comparison | **Mean difference** | **SE** | **Lower CI** | **Upper CI** | **t ratio** | **p value** |
|  | **T_pII_** | | | | | |
| *A. alpina* agg. – *D. octopetala* | -0.81 | 0.46 | -2.19 | 0.57 | -1.76 | 0.58 |
| *A. alpina* agg. – *R. ferrugineum* | -1.61 | 0.45 | -2.96 | -0.26 | -3.59 | **0.01** |
| *A. alpina* agg. – *R. ferrugineum I* | -0.80 | 0.39 | -1.99 | 0.38 | -2.03 | 0.40 |
| *A. alpina* agg. – *R. glacialis* | 0.66 | 0.39 | -0.51 | 1.84 | 1.69 | 0.62 |
| *A. alpina* agg. – *S. exarata* | -0.01 | 0.39 | -1.20 | 1.18 | -0.02 | 1.00 |
| *A. alpina* agg. – *V. vitis-idea* | -2.07 | 0.43 | -3.36 | -0.77 | -4.79 | **0.00** |
| *D. octopetala – R. ferrugineum* | -0.80 | 0.47 | -2.21 | 0.60 | -1.72 | 0.60 |
| *D. octopetala – R. ferrugineum I* | 0.01 | 0.42 | -1.25 | 1.26 | 0.01 | 1.00 |
| *D. octopetala – R. glacialis* | 1.47 | 0.41 | 0.23 | 2.71 | 3.56 | **0.01** |
| *D. octopetala – S. exarata* | 0.80 | 0.42 | -0.45 | 2.05 | 1.92 | 0.47 |
| *D. octopetala – V. vitis-idaea* | -1.26 | 0.45 | -2.61 | 0.10 | -2.79 | **0.09** |
| *R. ferrugineum – R. ferrugineumI* | 0.81 | 0.40 | -0.41 | 2.03 | 2.00 | 0.42 |
| *R. ferrugineum – R. glacialis* | 2.27 | 0.40 | 1.07 | 3.48 | 5.66 | **0.00** |
| *R. ferrugineum – S. exarata* | 1.60 | 0.40 | 0.39 | 2.82 | 3.96 | **0.00** |
| *R. ferrugineum – V. vitis-idaea* | -0.45 | 0.44 | -1.78 | 0.87 | -1.03 | 0.95 |
| *R. ferrugineum I – R. glacialis* | 1.47 | 0.34 | 0.44 | 2.49 | 4.29 | **0.00** |
| *R. ferrugineum – S. exarata* | 0.79 | 0.35 | -0.24 | 1.83 | 2.30 | 0.25 |
| *R. ferrugineum – V. vitis-idaea* | -1.26 | 0.39 | -2.42 | -0.10 | -3.27 | **0.02** |
| *R. glacialis – S. exarata* | -0.67 | 0.34 | -1.70 | 0.35 | -1.97 | 0.44 |
| *R. glacialis – V. vitis-idaea* | -2.73 | 0.38 | -3.88 | -1.58 | -7.13 | **0.00** |
| *S. exarata – V. vitis-idaea* | -2.06 | 0.39 | -3.22 | -0.90 | -5.33 | **0.00** |
| Comparison | **Mean difference** | **SE** | **Lower CI** | **Upper CI** | **t ratio** | **p value** |
|  | **T_init_** | | | | | |
| *A. alpina* agg. – *D. octopetala* | -1.56 | 0.77 | -4.03 | 0.91 | -2.03 | 0.42 |
| *A. alpina* agg. – *R. ferrugineum* | -3.30 | 0.77 | -5.77 | -0.83 | -4.30 | **0.00** |
| *A. alpina* agg. – *R. ferrugineum I* | -3.85 | 0.77 | -6.32 | -1.38 | -5.01 | **0.00** |
| *A. alpina* agg. – *R. glacialis* | -0.08 | 0.70 | -2.33 | 2.18 | -0.11 | 1.00 |
| *A. alpina* agg. – *S. exarata* | 0.66 | 0.77 | -1.80 | 3.13 | 0.86 | 0.97 |
| *A. alpina* agg. – *V. vitis-idea* | -3.66 | 0.73 | -6.00 | -1.32 | -5.02 | **0.00** |
| *D. octopetala – R. ferrugineum* | -1.74 | 0.77 | -4.21 | 0.73 | -2.27 | 0.30 |
| *D. octopetala – R. ferrugineum I* | -2.29 | 0.77 | -4.76 | 0.18 | -2.98 | 0.08 |
| *D. octopetala – R. glacialis* | 1.48 | 0.70 | -0.77 | 3.74 | 2.12 | 0.38 |
| *D. octopetala – S. exarata* | 2.22 | 0.77 | -0.24 | 4.69 | 2.89 | 0.10 |
| *D. octopetala – V. vitis-idaea* | -2.10 | 0.73 | -4.44 | 0.24 | -2.88 | 0.10 |
| *R. ferrugineum – R. ferrugineumI* | -0.55 | 0.77 | -3.02 | 1.92 | -0.72 | 0.99 |
| *R. ferrugineum – R. glacialis* | 3.23 | 0.70 | 0.97 | 5.48 | 4.60 | **0.00** |
| *R. ferrugineum – S. exarata* | 3.97 | 0.77 | 1.50 | 6.43 | 5.16 | **0.00** |
| *R. ferrugineum – V. vitis-idaea* | -0.36 | 0.73 | -2.70 | 1.99 | -0.49 | 1.00 |
| *R. ferrugineum I – R. glacialis* | 3.78 | 0.70 | 1.52 | 6.03 | 5.38 | **0.00** |
| *R. ferrugineum – S. exarata* | 4.52 | 0.77 | 2.05 | 6.98 | 5.88 | **0.00** |
| *R. ferrugineum – V. vitis-idaea* | 0.19 | 0.73 | -2.15 | 2.54 | 0.27 | 1.00 |
| *R. glacialis – S. exarata* | 0.74 | 0.70 | -1.51 | 2.99 | 1.05 | 0.94 |
| *R. glacialis – V. vitis-idaea* | -3.58 | 0.66 | -5.70 | -1.47 | -5.44 | **0.00** |
| *S. exarata – V. vitis-idaea* | -4.32 | 0.73 | -6.66 | -1.98 | -5.93 | **0.00** |
| Comparison | **Mean difference** | **SE** | **Lower CI** | **Upper CI** | **t ratio** | **p value** |
|  | **T_endo_** | | | | | |
| *A. alpina* agg. – *D. octopetala* | 1.13 | 0.55 | -0.62 | 2.87 | 2.07 | 0.40 |
| *A. alpina* agg. – *R. ferrugineum* | -0.63 | 0.55 | -2.37 | 1.12 | -1.15 | 0.91 |
| *A. alpina* agg. – *R. ferrugineum I* | 0.91 | 0.55 | -0.84 | 2.66 | 1.67 | 0.64 |
| *A. alpina* agg. – *R. glacialis* | -1.76 | 0.50 | -3.36 | -0.17 | -3.54 | **0.02** |
| *A. alpina* agg. – *S. exarata* | -5.36 | 0.52 | -7.01 | -3.70 | -10.35 | **0.00** |
| *A. alpina* agg. – *V. vitis-idea* | -2.19 | 0.52 | -3.85 | -0.54 | -4.24 | **0.00** |
| *D. octopetala – R. ferrugineum* | -1.75 | 0.55 | -3.50 | -0.01 | -3.21 | 0.05 |
| *D. octopetala – R. ferrugineum I* | -0.22 | 0.55 | -1.96 | 1.53 | -0.40 | 1.00 |
| *D. octopetala – R. glacialis* | -2.89 | 0.50 | -4.48 | -1.30 | -5.80 | **0.00** |
| *D. octopetala – S. exarata* | -6.48 | 0.52 | -8.14 | -4.83 | -12.53 | **0.00** |
| *D. octopetala – V. vitis-idaea* | -3.32 | 0.52 | -4.98 | -1.66 | -6.41 | **0.00** |
| *R. ferrugineum – R. ferrugineumI* | 1.54 | 0.55 | -0.21 | 3.28 | 2.81 | 0.11 |
| *R. ferrugineum – R. glacialis* | -1.14 | 0.50 | -2.73 | 0.46 | -2.28 | 0.29 |
| *R. ferrugineum – S. exarata* | -4.73 | 0.52 | -6.39 | -3.08 | -9.14 | **0.00** |
| *R. ferrugineum – V. vitis-idaea* | -1.57 | 0.52 | -3.22 | 0.09 | -3.03 | 0.07 |
| *R. ferrugineum I – R. glacialis* | -2.67 | 0.50 | -4.27 | -1.08 | -5.37 | **0.00** |
| *R. ferrugineum – S. exarata* | -6.27 | 0.52 | -7.92 | -4.61 | -12.11 | **0.00** |
| *R. ferrugineum – V. vitis-idaea* | -3.10 | 0.52 | -4.76 | -1.45 | -5.99 | **0.00** |
| *R. glacialis – S. exarata* | -3.60 | 0.47 | -5.09 | -2.10 | -7.70 | **0.00** |
| *R. glacialis – V. vitis-idaea* | -0.43 | 0.47 | -1.93 | 1.06 | -0.92 | 0.97 |
| *S. exarata – V. vitis-idaea* | 3.17 | 0.49 | 1.60 | 4.73 | 6.49 | **0.00** |
| Comparison | **Mean difference** | **SE** | **Lower CI** | **Upper CI** | **t ratio** | **p value** |
|  | **T_exo_** | | | | | |
| *A. alpina* agg. – *D. octopetala* | 0.69 | 0.67 | -1.39 | 2.77 | 1.03 | 0.90 |
| *A. alpina* agg. – *R. ferrugineum* | -0.22 | 0.67 | -2.30 | 1.86 | -0.32 | 1.00 |
| *A. alpina* agg. – *R. ferrugineum I* | 1.08 | 0.61 | -0.82 | 2.97 | 1.76 | 0.51 |
| *A. alpina* agg. – *R. glacialis* | -0.04 | 0.61 | -1.94 | 1.85 | -0.07 | 1.00 |
| *A. alpina* agg. – *V. vitis-idea* | -0.79 | 0.64 | -2.77 | 1.18 | -1.25 | 0.81 |
| *D. octopetala – R. ferrugineum* | -0.91 | 0.67 | -2.99 | 1.17 | -1.35 | 0.75 |
| *D. octopetala – R. ferrugineum I* | 0.39 | 0.61 | -1.51 | 2.28 | 0.63 | 0.99 |
| *D. octopetala – R. glacialis* | -0.73 | 0.61 | -2.63 | 1.16 | -1.20 | 0.83 |
| *D. octopetala – V. vitis-idaea* | -1.48 | 0.64 | -3.46 | 0.49 | -2.33 | 0.22 |
| *R. ferrugineum – R. ferrugineumI* | 1.29 | 0.61 | -0.61 | 3.19 | 2.11 | 0.32 |
| *R. ferrugineum – R. glacialis* | 0.17 | 0.61 | -1.72 | 2.07 | 0.28 | 1.00 |
| *R. ferrugineum – V. vitis-idaea* | -0.58 | 0.64 | -2.55 | 1.40 | -0.91 | 0.94 |
| *R. ferrugineum I – R. glacialis* | -1.12 | 0.55 | -2.82 | 0.58 | -2.04 | 0.35 |
| *R. ferrugineum – V. vitis-idaea* | -1.87 | 0.57 | -3.65 | -0.09 | -3.26 | 0.04 |
| *R. glacialis – V. vitis-idaea* | -0.75 | 0.57 | -2.53 | 1.03 | -1.31 | 0.78 |
| Comparison | **Mean difference** | **SE** | **Lower CI** | **Upper CI** | **t ratio** | **p value** |
|  | **LT_10_** | | | | | |
| *A. alpina* agg. – *D. octopetala* | -1.93 | 0.07 | -2.14 | -1.72 | -26.98 | **0.00** |
| *A. alpina* agg. – *R. ferrugineum* | 1.57 | 0.07 | 1.36 | 1.78 | 21.91 | **0.00** |
| *A. alpina* agg. – *R. ferrugineum I* | 1.48 | 0.07 | 1.27 | 1.69 | 20.70 | **0.00** |
| *A. alpina* agg. – *R. glacialis* | 3.15 | 0.07 | 2.94 | 3.36 | 44.03 | **0.00** |
| *A. alpina* agg. – *S. exarata* | 0.41 | 0.07 | 0.20 | 0.62 | 5.68 | **0.00** |
| *A. alpina* agg. – *V. vitis-idea* | -3.18 | 0.07 | -3.40 | -2.97 | -44.13 | **0.00** |
| *D. octopetala – R. ferrugineum* | 3.50 | 0.07 | 3.29 | 3.71 | 48.89 | **0.00** |
| *D. octopetala – R. ferrugineum I* | 3.41 | 0.07 | 3.20 | 3.62 | 47.68 | **0.00** |
| *D. octopetala – R. glacialis* | 5.08 | 0.07 | 4.87 | 5.29 | 71.01 | **0.00** |
| *D. octopetala – S. exarata* | 2.34 | 0.07 | 2.13 | 2.55 | 32.66 | **0.00** |
| *D. octopetala – V. vitis-idaea* | -1.25 | 0.07 | -1.47 | -1.04 | -17.38 | **0.00** |
| *R. ferrugineum – R. ferrugineumI* | -0.09 | 0.07 | -0.30 | 0.12 | -1.21 | **0.89** |
| *R. ferrugineum – R. glacialis* | 1.58 | 0.07 | 1.37 | 1.79 | 22.12 | **0.00** |
| *R. ferrugineum – S. exarata* | -1.16 | 0.07 | -1.37 | -0.95 | -16.22 | **0.00** |
| *R. ferrugineum – V. vitis-idaea* | -4.75 | 0.07 | -4.96 | -4.54 | -65.86 | **0.00** |
| *R. ferrugineum I – R. glacialis* | 1.67 | 0.07 | 1.46 | 1.88 | 23.33 | **0.00** |
| *R. ferrugineum – S. exarata* | -1.07 | 0.07 | -1.29 | -0.86 | -15.02 | **0.00** |
| *R. ferrugineum – V. vitis-idaea* | -4.66 | 0.07 | -4.88 | -4.45 | -64.67 | **0.00** |
| *R. glacialis – S. exarata* | -2.74 | 0.07 | -2.95 | -2.53 | -38.35 | **0.00** |
| *R. glacialis – V. vitis-idaea* | -6.33 | 0.07 | -6.55 | -6.12 | -87.81 | **0.00** |
| *S. exarata – V. vitis-idaea* | -3.59 | 0.07 | -3.80 | -3.38 | -49.77 | **0.00** |
| Comparison | **Mean difference** | **SE** | **Lower CI** | **Upper CI** | **t ratio** | **p value** |
|  | LT_50_ | | | | | |
| *A. alpina* agg. – *D. octopetala* | 0.63 | 0.07 | 0.43 | 0.84 | 9.14 | **0.00** |
| *A. alpina* agg. – *R. ferrugineum* | 4.33 | 0.07 | 4.13 | 4.54 | 62.57 | **0.00** |
| *A. alpina* agg. – *R. ferrugineum I* | 4.54 | 0.07 | 4.34 | 4.75 | 65.63 | **0.00** |
| *A. alpina* agg. – *R. glacialis* | 5.54 | 0.07 | 5.34 | 5.74 | 80.03 | **0.00** |
| *A. alpina* agg. – *S. exarata* | 2.80 | 0.07 | 2.60 | 3.01 | 40.50 | **0.00** |
| *A. alpina* agg. – *V. vitis-idea* | -2.29 | 0.07 | -2.50 | -2.09 | -32.86 | **0.00** |
| *D. octopetala – R. ferrugineum* | 3.70 | 0.07 | 3.49 | 3.90 | 53.43 | **0.00** |
| *D. octopetala – R. ferrugineum I* | 3.91 | 0.07 | 3.71 | 4.11 | 56.49 | **0.00** |
| *D. octopetala – R. glacialis* | 4.91 | 0.07 | 4.70 | 5.11 | 70.90 | **0.00** |
| *D. octopetala – S. exarata* | 2.17 | 0.07 | 1.97 | 2.37 | 31.36 | **0.00** |
| *D. octopetala – V. vitis-idaea* | -2.93 | 0.07 | -3.13 | -2.72 | -41.92 | **0.00** |
| *R. ferrugineum – R. ferrugineumI* | 0.21 | 0.07 | 0.01 | 0.42 | 3.06 | **0.04** |
| *R. ferrugineum – R. glacialis* | 1.21 | 0.07 | 1.00 | 1.41 | 17.46 | **0.00** |
| *R. ferrugineum – S. exarata* | -1.53 | 0.07 | -1.73 | -1.32 | -22.07 | **0.00** |
| *R. ferrugineum – V. vitis-idaea* | -6.62 | 0.07 | -6.83 | -6.42 | -94.92 | **0.00** |
| *R. ferrugineum I – R. glacialis* | 1.00 | 0.07 | 0.79 | 1.20 | 14.41 | **0.00** |
| *R. ferrugineum – S. exarata* | -1.74 | 0.07 | -1.94 | -1.54 | -25.13 | **0.00** |
| *R. ferrugineum – V. vitis-idaea* | -6.84 | 0.07 | -7.04 | -6.63 | -97.95 | **0.00** |
| *R. glacialis – S. exarata* | -2.74 | 0.07 | -2.94 | -2.53 | -39.54 | **0.00** |
| *R. glacialis – V. vitis-idaea* | -7.83 | 0.07 | -8.04 | -7.63 | -112.24 | **0.00** |
| *S. exarata – V. vitis-idaea* | -5.10 | 0.07 | -5.30 | -4.89 | -73.03 | **0.00** |

**Supplemental Table 5. Pearson correlation of the measured parameters.** Boxes indicate the group of parameters obtained using the same method. Correlations between T-F0 and DSC parameters were obtained by using paramters obtained from the same twig/ stalk or leaf rosette, respectively. Mean values were used to correlate parameters with LT_10_ and LT_50_. Pearson correlation was applied. Correlations above 0.5 are given in bold.

|  | T-F0 parameter | | | | DSC parameter | | | | Tissue test | | |
| --- | --- | --- | --- | --- | --- | --- | --- | --- | --- | --- | --- |
|  | **T_c_** | **T_p_** | **T_pII_** | **T_init_** | | **T_endo_** | **T_exo_** | **mLT_10_** | | **mLT_50_** |  |
| T_c_ | 1 | **0.59** | 0.46 | 0.23 | | -0.38 | 0.26 | **0.74** | | **0.72** |  |
| T_p_ |  | 1 | **0.64** | **0.51** | | -0.26 | 0.35 | **0.92** | | **0.79** |  |
| T_pII_ |  |  | **1** | **0.68** | | -0.26 | **0.57** | **0.60** | | **0.46** |  |
| T_init_ |  |  |  | 1 | | -0.33 | 0.26 | 0.25 | | 0.10 |  |
| T_endo_ |  |  |  |  | | 1 | **0.73** | -0.03 | | 0.00 |  |
| T_exo_ |  |  |  |  | |  | 1 | 0.27 | | 0.43 |  |
| mLT_10_ |  |  |  |  | |  |  | 1 | | **0.94** |  |
| mLT_50_ |  |  |  |  | |  |  |  | | 1 |  |

**Supplemental Table 6. Species specific pearson correlation of the T-F0 and DSC parameters.** Correlations between T-F0 and DSC parameters were obtained by using paramters obtained from the same twig/ stalk or leaf rosette, respectively. Pearson correlation was applied. Correlations above 0.5 are given in bold. *T_exo_ could not be determined in *S. exarata.*

|  | **T-F0 parameter** | | | **DSC parameter** | | |
| --- | --- | --- | --- | --- | --- | --- |
| *R. glacialis* | **T_c_** | **T_p_** | **T_pII_** | **T_init_** | **T_endo_** | **T_exo_** |
| **T_c_** | 1.00 | **0.57** | **0.53** | 0.09 | **-0.63** | **-0.86** |
| **T_p_** |  | 1.00 | **0.70** | -0.10 | **0.74** | 0.48 |
| **T_pII_** |  |  | 1.00 | **-0.56** | **0.77** | **0.60** |
| **T_init_** |  |  |  | 1.00 | 0.04 | 0.29 |
| **T_endo_** |  |  |  |  | 1.00 | **0.90** |
| **T_exo_** |  |  |  |  |  | 1.00 |
| ***S. exarata*** | **T_c_** | **T_p_** | **T_pII_** | **T_init_** | **T_endo_** |  |
| **T_c_** | 1.00 | **0.60** | 0.42 | **-0.69** | **0.68** | na* |
| **T_p_** |  | 1.00 | **0.81** | **0.61** | **-0.72** |  |
| **T_pII_** |  |  | 1.00 | **0.74** | **-0.82** |  |
| **T_init_** |  |  |  | 1.00 | **-0.94** |  |
| **T_endo_** |  |  |  |  | 1.00 |  |
| ***A. alpina* agg.** | **T_c_** | **T_p_** | **T_pII_** | **T_init_** | **T_endo_** | **T_exo_** |
| **T_c_** | 1.00 | **0.58** | **0.65** | 0.15 | **0.69** | 0.45 |
| **T_p_** |  | 1.00 | **0.55** | **0.92** | **0.50** | **0.73** |
| **T_pII_** |  |  | 1.00 | **0.65** | **0.97** | **0.88** |
| **T_init_** |  |  |  | 1.00 | **0.76** | **0.93** |
| **T_endo_** |  |  |  |  | 1.00 | **0.95** |
| **T_exo_** |  |  |  |  |  | 1.00 |
| ***R. ferrugineum I*** | **T_c_** | **T_p_** | **T_pII_** | **T_init_** | **T_endo_** | **T_exo_** |
| **T_c_** | 1.00 | **0.59** | **0.70** | -0.20 | -0.06 | 0.30 |
| **T_p_** |  | 1.00 | **0.52** | **0.67** | **0.74** | **0.63** |
| **T_pII_** |  |  | 1.00 | 0.16 | 0.30 | **0.86** |
| **T_init_** |  |  |  | 1.00 | **0.99** | **0.79** |
| **T_endo_** |  |  |  |  | 1.00 | **0.86** |
| **T_exo_** |  |  |  |  |  | 1.00 |
| ***R. ferrugineum*** | **T_c_** | **T_p_** | **T_pII_** | **T_init_** | **T_endo_** | **T_exo_** |
| **T_c_** | 1.00 | -0.19 | 0.22 | -0.35 | 0.28 | **-0.58** |
| **T_p_** |  | 1.00 | -0.29 | **0.66** | 0.28 | **0.86** |
| **T_pII_** |  |  | 1.00 | **0.81** | **0.59** | 0.08 |
| **T_init_** |  |  |  | 1.00 | 0.31 | 0.34 |
| **T_endo_** |  |  |  |  | 1.00 | **0.59** |
| **T_exo_** |  |  |  |  |  | 1.00 |
| ***D.octopetala*** | **T_c_** | **T_p_** | **T_pII_** | **T_init_** | **T_endo_** | **T_exo_** |
| **T_c_** | 1.00 | 0.34 | 0.00 | **-0.91** | **0.63** | **0.56** |
| **T_p_** |  | 1.00 | **0.51** | **-0.67** | **0.90** | **0.87** |
| **T_pII_** |  |  | 1.00 | -1.00 | **-0.99** | -0.27 |
| **T_init_** |  |  |  | 1.00 | -0.29 | -0.26 |
| **T_endo_** |  |  |  |  | 1.00 | **0.98** |
| **T_exo_** |  |  |  |  |  | 1.00 |
| ***V****.* ***vitis-idaea*** | **T_c_** | **T_p_** | **T_pII_** | **T_init_** | **T_endo_** | **T_exo_** |
| **T_c_** | 1.00 | **0.68** | 0.29 | 0.45 | 0.49 | **0.56** |
| **T_p_** |  | 1.00 | 0.23 | **0.53** | **0.75** | **0.70** |
| **T_pII_** |  |  | 1.00 | -0.20 | **0.81** | **0.86** |
| **T_init_** |  |  |  | 1.00 | 0.27 | -0.22 |
| **T_endo_** |  |  |  |  | 1.00 | **0.74** |
| **T_exo_** |  |  |  |  |  | 1.00 |
